# Gender Differences in Predictors of Left Ventricular Myocardial Relaxation in Non-Obese, Healthy Individuals

**DOI:** 10.1371/journal.pone.0125107

**Published:** 2015-04-30

**Authors:** Haruki Sekiguchi, Ken Shimamoto, Naoki Sekiguchi, Yuri Ozaki, Kaoru Shimizu, Yufuko Takahashi, Akiko Sakai, Fujio Tatsumi, Naoko Ishizuka, Masatoshi Kawana

**Affiliations:** 1 Department of Cardiology, Aoyama Hospital Tokyo Women’s Medical University, Tokyo, Japan; 2 Department of Cardiology, Tokyo Women’s Medical University, Tokyo, Japan; 3 Department of Cardiology, National Hospital Organization Yokohama Medical Center, Kanagawa, Japan; Inserm, FRANCE

## Abstract

**Background:**

Previous studies indicate that individuals with metabolic syndrome (MetS) might be at risk for left ventricular (LV) diastolic dysfunction. However, little is known about which metabolic factors contribute to the development of LV dysfunction in individuals who are not obese or overweight and who do not have diabetes mellitus and/or cardiovascular disease.

**Methods:**

Participants without diabetes mellitus, systolic dysfunction, or other heart diseases underwent a thorough physical examination, including tissue Doppler echocardiography. A peak early mitral annular velocity (e′) of <5.0 was designated as indicating abnormal LV myocardial relaxation (LVMR). We performed single and multiple logistic regression analyses of e′ and cardiovascular risk factors, including MetS factors and indicators of major organ dysfunction. Normal-weight subjects (body mass index <25 kg/m^2^) were also analyzed.

**Results:**

A total of 1055 individuals (mean age, 63 ± 13 years) participated, of which 307 (29.1%) had MetS and 199 (18.9%) had abnormal LVMR. Multiple logistic regression analysis revealed waist circumference (WC) (odds ratio [OR] 1.04, P < 0.05) and age (OR 1.10, P < 0.05) to be predictors of abnormal LVMR. In normal-weight subjects (n = 806), aging (OR 1.08, P < 0.01), abnormal WC (OR 3.80, P < 0.01), and renal dysfunction (OR 2.14, P < 0.01) were predictors of abnormal LVMR. Among MetS factors, abnormal WC in men (OR 3.70, P < 0.01) and high diastolic blood pressure (DBP) in women (OR 4.00, P = 0.01) were related to abnormal LVMR.

## Introduction

Heart failure is a progressive condition, and its incidence increases with advanced age [1–3]. Previous studies have reported that heart failure with a normal ejection fraction (EF) often occurs at a higher frequency in older women than in older men [4, 5]. Echocardiographic classification of diastolic function in cross-sectional community studies has shown diastolic dysfunction to be highly prevalent and associated with heart failure [6, 7] and heart failure prognosis [8].

Obesity is a well-known reliable predictor of heart failure in middle-aged and elderly subjects [1]. However, a recent systematic review and meta-analysis revealed that body mass index (BMI) is not a risk factor of cardiovascular disease in metabolically healthy normal-weight subjects (BMI < 25 kg/m^2^). However, obesity in metabolically healthy subjects is associated with increased mortality and cardiovascular death [9]. Metabolic syndrome (MetS) is a well-described constellation of risk factors, such as high blood pressure, high cholesterol, and central adiposity, that leads to the development of cardiovascular disease [10]. The degree of MetS clustering is strongly related to left ventricular (LV) mass [11], wall thickness [12], and mitral E/A ratio (i.e., the ratio of early to late diastolic flow velocities on Doppler) [13], and patients with MetS are thought to have a risk of heart failure [14]. The progressive addition of MetS risk factors, such as obesity [15], diabetes, and/or dyslipidemia, is associated with increased LV mass, independently of hypertension [16]. In a previous study, Canepa et al. (from the Baltimore Longitudinal Study of Aging) graded diastolic function in a population-based sample and found waist circumference (WC) to be predictive of abnormal diastolic function [17]. Their findings revealed that the assessment of diastolic dysfunction without tissue Doppler imaging (TDI) had limited accuracy because of the pseudo-normalization that occurs in two-dimensional echocardiography. Therefore, TDI technology has become a standard method of assessing LV diastolic function, providing both pathophysiological and prognostic insight into systolic and diastolic heart failure [18, 19]. Although recent studies have reported a relationship between age and LV myocardial relaxation (LVMR) according to gender [20], the association of MetS and its component criteria on cardiac structure and function is poorly characterized. Moreover, to the best of our knowledge, no previous reports have evaluated the relationship between LVMR and MetS risk factors by TDI among healthy individuals without diabetes mellitus and/or cardiovascular disease.

Age-related changes in LV diastolic indices have been previously reported in a community-based study [21]. However, gender-specific differences in LV diastolic indices remain poorly understood in healthy subjects. In addition to MetS risk factors, other factors, such as aging and BMI, have been reported as risk factors of LVMR [22, 23]. However, no previous report has evaluated cardiovascular, MetS, and organ dysfunction risk factors among healthy individuals according to gender using multiple regression analysis.

Total Health Care (THC) is a health check system offered by our institution that includes over 1,000 registered members. We conduct detailed annual health examinations, which comprise detailed metabolic profiles, two-dimensional and TDI echocardiography, pulmonary functional tests, chest computed tomography scans, head magnetic resonance imaging/angiography, gastrointestinal endoscopy, and abdominal ultrasonography. Registered members are monitored for several years and have been followed for a mean 52 months.

In the present study, we analyzed the relationship between LV diastolic dysfunction and risk factors of MetS and cardiovascular disease in healthy individuals based on data obtained from the records of our unique THC system.

## Materials and Methods

### Subjects and protocol

Participants were registered THC members who underwent annual medical examinations between 2004 and 2011. Medical histories and physical examination findings were recorded for all participants, and participants underwent the following examinations on the first and second visits, which were within 6 month of registration: (1) Tissue Doppler echocardiography was conducted to assess LV diastolic function. (2) Blood pressure was measured after a 10-min rest in the sitting position and was expressed as the mean of three consecutive measurements in each arm using the conventional cuff method. (3) Blood sampling was performed in the morning after a 12-h overnight fast. The following blood components were subsequently assessed by standard laboratory techniques: plasma triglycerides (TG), high-density lipoprotein cholesterol (HDL-C), low-density lipoprotein cholesterol (LDL-C), fasting plasma glucose (FPG), hemoglobin (Hb), serum albumin concentration (Alb), uric acid (UA), C-reactive protein (CRP), and serum creatinine (Cr). (4) Pulmonary functional tests were performed to evaluate the following parameters: the ratio of forced expiratory volume in 1 s (FEV1) and forced vital capacity (VC; expressed as a percent predicted for age and sex). The annual health examination items are listed in Table A in S1 File.

Exclusion criteria included the following: 1) history or findings of cardiovascular disease, including heart failure symptoms or systolic dysfunction (LV ejection fraction [LVEF] of <55%), coronary artery disease, significant valvular heart disease (i.e., greater than mild valvular insufficiency or stenosis), and/or hypertrophic cardiomyopathy; 2) diabetes mellitus; 3) current pregnancy or lactation; and/or 4) major systemic illness such as malignancy.

### Ethics statement

The Human Research Protection Office of Tokyo Women’s Medical University approved this protocol (#2507). Written informed consent was obtained from all participants prior to study enrollment.

### Definition of MetS

We used the following definition of MetS: (i) WC ≥85 cm for males or ≥90 cm for females and two or more of the following four disorders: (ii) serum triglyceride ≥150 mg/dL, (iii) high-density lipoprotein cholesterol <40 mg/dL, (iv) systolic blood pressure (SBP) ≥130 mmHg and/or diastolic BP ≥85 mmHg, and (v) fasting blood glucose ≥110 mg/dL. These are the criteria for MetS as proposed by the Japanese Society of Hypertension and seven additional societies in 2005 [24, 25].

### Echocardiographic examination

Echocardiographic examination was performed with commercially available equipment (Vivid 7, GE Healthcare UK Ltd, Amersham Place, England) using a broadband transducer (S3). Two experienced sonographers performed the echocardiographic examinations and data collection. Both were free to access clinical information but were blinded to the study protocol.

### Definition of cardiac dysfunction

EF was measured by quantitative two-dimensional echocardiography, as previously reported [8]. Systolic dysfunction was defined as an LVEF of <55%. Diastolic function was assessed by pulse-wave Doppler examination of mitral flow and Doppler imaging of the medial mitral annulus according to Redfield et al. [8]. In brief, TDI was performed in the apical 4-chamber view. Pulsed wave Doppler was used to obtain mitral annular velocity on the septal side. Peak early mitral annular velocity (e′) and peak late mitral annular velocity (a′) were recorded and measured; e′ was used as an index of LVMR [20], An abnormal, severe LVMR was defined as an e′ value of <5 m/s according to the classification previously described by Lester et al. [26].

### Extra-cardiac major organ system dysfunction

Extra-cardiac major organ system dysfunction was evaluated as a risk factor for diastolic dysfunction. The measurement variables defining non-cardiac dysfunction included the following: 1) renal dysfunction (creatinine clearance [Ccr] <60 mL/min), 2) hyperuricemia (UA >7.0 mg/dL), 3) hypoalbuminemia (Alb <3.5 mg/dL), 4) anemia (Hb <12 mg/dL in men, Hb <11 mg/dL in women), 5) pulmonary dysfunction (%VC <80% or FEV1.0 <70%), and 6) inflammation (CRP >0.03 mg/dL).

### Statistical analysis

Prior to analysis, we reviewed the distribution of the data using the Kolmogorov-Smirnov test for each pair. Single correlations between e´ and various potential risk factors (i.e., age, BMI, WC, TG, HDL-C, LDL-C, FPG, Hb, SBP, diastolic blood pressure [DBP], HbA1c, UA, Alb, Ccr, CRP, FEV1 and %VC, RWT, LVMI, or EF) were assessed using Spearman’s rank correlation coefficients when the values were not normally distributed or Pearson’s correlation coefficient when the values were normally distributed. Continuous and categorical data were analyzed using the *t*-test and chi-square test, respectively.

Univariate or multivariable logistic-regression models were applied to e′o(<5 m/s) and the following parameters: age, BMI, WC ≥90 cm in women, WC ≥85 cm in men, TG ≥150 mg/dL, HDL-C ≤40 mg/dL, LDL-C ≥140 mg/dL, FPG ≥110 mg/dL, SBP ≥135 mmHg, DBP ≥85 mmHg, renal dysfunction, hyperuricemia, hypoalbuminemia, anemia, inflammation, and pulmonary dysfunction. A multivariable logistic regression model that included potential factors identified as significant (*P* < 0.25) by univariate analysis was applied. Factors were also analyzed according to gender. Moreover, in a sub-group analysis, we analyzed the study population as healthy individuals (n = 806) who were not obese (BMI < 30 kg/m^2^) or overweight (BMI < 25 kg/m^2^).

Normally distributed data are reported as means ± standard deviation (SD), non-normally distributed data as medians and interquartile range, and categorical data as absolute values and percentages. A *P* value of <0.05 was considered to indicate statistical significance unless otherwise stated. All statistical analyses were performed using SPSS statistics (version 22.0, SPSS Institute Inc., Chicago, IL).

## Results

### Baseline characteristics

In total, 1278 consecutive individuals registered for a health examination between 2004 and 2011. Following the exclusion of individuals with diabetes mellitus (n = 188), systolic dysfunction (n = 24), and other heart diseases (n = 11), 1055 healthy individuals with a mean age of 63 ± 12.8 years (range, 22–98 years) were included in the study. The follow-up rate was 96%. Of the study population, 249 (23.6%) subjects were obese or overweight (BMI ≥25 kg/m^2^), and 307 (29.1%) were observed to have MetS. Moreover, 21.0% of men (130/620) and 15.9% of women (49/435) had abnormal LVMR. The demographic and clinical characteristics of the study sample showed the mean values of all data were within standard values (Table 1). The echocardiography characteristics by gender showed that the study population had a normal EF; the echocardiography data were comparable to those of a previous report (S1 File—Table B) [20].

**Table 1:** Baseline characteristics.

Characteristic	All	Male	Female	All	Male	Female
Demographic and anthropometric characteristics	All participants			Normal weight(BMI <25 kg/m^2^)		
N	1055	620	435	806	421	385
Age, yr	62.8 ± 12.8	63.2 ± 12.8	62.2 ± 12.7	62.8±12.9	63.6 ± 13.0	61.9 ± 12.7
Men, n (%)	619 (58.7)	N.A.	N.A.	421 (52.2)	N.A.	N.A.
Body mass index, kg/m^2^	22.8 ± 3.2	23.9 ± 2.9	21.1 ± 2.9	21.4±2.1	22.4 ± 1.8	20.4 ± 2.1
Metabolic syndrome, n (%)	307 (29.1)	273 (44.0)	34 (7.8)	141 (17.5)	127 (30.2)	14 (3.3)
**Metabolic syndrome risk factors**						
Waist circumference, cm	84 ± 10	88 ± 8	80 ± 9	82 ± 8	84 ± 6	78 ± 8
Systolic blood pressure, mmHg	120 ± 16	124 ± 16	116 ± 16	119 ± 16	123 ± 16	115 ± 16
Diastolic blood pressure, mmHg	73 ± 11	75 ± 11	70 ± 10	72 ± 10	74 ± 10	69 ± 10
TG, mg/dL	113 ± 81	129 ± 95	90 ± 47	105 ± 79	119 ± 97	88 ± 47
HDL-C, mg/dL	63 ± 18	57 ± 14	71 ± 18	66 ± 18	59 ± 15	73 ± 18
FPG, mg/dL	100 ± 10	103 ± 10	96 ± 10	99 ± 10	101 ± 10	96 ± 10
**Other cardiovascular risk factors**						
LDL-C, mg/dL	123 ± 30	121 ± 29	126 ± 32	123 ± 31	120 ± 30	125 ± 32
HbA1c, %	5.2 ± 0.3	5.2 ± 0.3	5.1 ± 0.3	5.1 ± 0.3	5.1 ± 0.3	5.1 ± 0.3
UA, mg/dL	5.6 ± 1.4	6.3 ± 1.2	4.7 ± 1.0	5.4 ± 1.3	6.2 ± 1.2	4.7 ± 1.0
Serum albumin, g/dL	4.6 ± 0.3	4.6 ± 0.4	4.5 ± 0.5	4.6 ± 0.3	4.6 ± 0.3	4.6 ± 0.3
Hemoglobin concentration, g/dL	14.1 ± 1.5	14.7 ± 1.4	13.2 ± 1.0	13.9 ± 4.2	14.6 ± 1.5	13.2 ± 1.0
Ccr, mL/min	78 ± 30	77 ± 33	80 ± 24	80 ± 26	83 ± 29	77 ± 22
CRP, mg/dL	0.07 ± 0.08	0.08 ± 0.08	0.06 ± 0.08	0.07 ± 0.08	0.07 ± 0.08	0.06 ± 0.08
%VC	108 ± 16	107 ± 16	109 ± 16	109 ± 16	108 ± 17	109 ± 16
FEV1.0%	80 ± 7	79 ± 7	81 ± 7	80 ± 7	78 ± 8	81 ± 7

Abbreviations: TG, triglyceride; HDL-C, high-density lipoprotein cholesterol; FPG, fasting plasma glucose; LDL-C, low-density lipoprotein cholesterol; HbA1c, hemoglobin A1c; UA, uric acid; Ccr, creatinine clearance; CRP, C-reactive protein; VC, forced vital capacity; FEV1.0%, ratio of forced expiratory volume in 1 s

### Predictors of abnormal LVMR among healthy participants

In the analysis of single correlations between e′ and various potential risk factors involving continuous data, e′ was negatively correlated with age, BMI, WC, SBP, DBP, TG, FPG, and Ccr and positively correlated with HDL-C, Alb, %VC, and FEV1. No correlation was observed with Hb or LDL-C (Table 2). Next, various factors potentially contributing to abnormal LVMR (e′ <5 m/s) were evaluated. Significant differences in age, gender, WC, SBP, HDL-C, FPG, HbA1c, UA, Ccr, and %VC were observed between the normal and abnormal LVMR groups. However, no association was found between abnormal LVMR and DBP (*P* = 0.2307), TG (*P* = 0.4157), LDL-C (*P* = 0.0549), Hb (*P* = 0.3717), or FEV1.0 (*P* = 0.0634) in all subjects. Meanwhile, in normal-weight individuals, the same differences were observed between the normal LVMR and the abnormal LVMR groups (Table 3).

**Table 2:** Spearman’s rank correlation coefficients between abnormal left ventricular myocardial relaxation (LVMR) and various potential risk factors.

	All participants		Normal weight (BMI <25 kg/m^2^)	
	Correlation coefficient	P value	Correlation coefficient	P value
Age	-0.567	<0.001	-0.593	<0.001
Body mass index	-0.288	<0.001	-0.252	<0.001
Waist circumference	-0.327	<0.001	-0.304	<0.001
Systolic blood pressure	-0.347	<0.001	-0.362	<0.001
Diastolic blood pressure	-0.157	<0.001	-0.151	<0.001
HDL-C	0.155	<0.001	0.128	<0.001
Triglyceride	-0.160	<0.001	-0.126	<0.001
Fast plasma glucose	-0.211	<0.001	-0.220	<0.001
Serum albumin	0.069	<0.001	0.079	<0.001
UA	-0.192	<0.001	-0.181	<0.001
%VC	0.190	<0.001	0.188	<0.001
FEV1.0	0.167	<0.001	0.185	<0.001
Ccr	-0.199	<0.001	-0.368	<0.001
Hemoglobin		NS		NS
LDL-C		NS		NS

Abbreviations: HDL-C, high-density lipoprotein cholesterol; UA, uric acid; VC, forced vital capacity; FEV1.0%, ratio of forced expiratory volume in 1 s; Ccr, creatinine clearance; LDL-C, low-density lipoprotein cholesterol

**Table 3:** Predictors of abnormal LVMR.

		All participants		Normal weight (BMI <25 kg/m^2^)		
Factor	Normal LVMR	Abnormal LVMR	P value	Normal LVMR	Abnormal LVMR	P value
n	856	199		665	141	
Age, yr	60.5 ± 12.4	72.4 ± 9.7	<0.001	60.6 ± 12.4	73.0 ± 10.0	<0.001
Men, n (%)	491 (57.3)	130 (65.3)	0.0473	294 (44.2)	127 (90.0)	0.021
Age < 65 yr	524 (61.2)	36 (18.1)		403 (60.6)	24 (17.0)	
Age ≥ 65 yr	332 (38.8)	163 (81.9)		262 (39.4)	117 (83.0)	
Body mass index, kg/m^2^	22.5 ± 3.2	23.7 ± 3.1	<0.001	21.3 ± 2.2	22.2 ± 1.9	<0.001
**Metabolic syndrome risk factors**						
Waist circumference, cm	83.6 ± 9.5	87.8 ± 8.8	<0.001	80.3 ± 7.5	84.3 ± 7.0	<0.001
SBP, mmHg	119 ± 16	126 ± 15	<0.001	118 ± 16	125 ± 15	<0.001
DBP, mmHg	72.5 ± 10.5	73.5 ± 10.9	0.2307	71 ± 10	72 ± 10	0.402
TG, mg/dL	112.0 ± 81.4	117.2 ± 78.9	0.4154	113± 79	110 ± 81	0.399
HDL-C, mg/dL	63.7 ± 18.1	59.4 ± 15.0	0.002	67 ± 18	61 ± 15	< 0.001
FPG, mg/dL	99.4 ± 10.1	102.7 ± 9.9	<0.0001	98 ± 9	101 ± 10	< 0.001
**Other cardiovascular risk factors**						
LDL-C, mg/dL	123.9 ± 30.9	119.3 ± 27.9	0.0549	123± 31	120 ± 28	0.178
HbA1c,%	5.6 ± 1.4	6.0 ± 1.3	0.0003	5.3 ± 1.3	5.8 ± 1.2	<0.001
UA, mg/dL	5.6 ± 1.4	6.0 ± 1.3	0.0003	5.3 ± 1.3	5.8 ± 1.2	<0.001
Serum albumin, g/dL	4.6 ± 0.3	4.5 ± 0.3	<0.0001	4.6 ± 0.3	4.5 ± 0.3	0.056
Hemoglobin concentration, g/dL	14.1 ± 1.4	14.0 ± 1.5	0.3717	14.0 ± 1.4	13.7 ± 1.5	0.1000
Ccr, mL/min	88.9 ± 29.0	70.2 ± 25.3	<0.0001	83.3 ± 24.8	65.1 ± 24.6	<0.001
%VC	109 ± 16	102 ± 15	<0.0001	110 ± 16	102 ± 16	<0.001
FEV1.0%	80 ± 7	79 ± 6	0.0634	80 ± 7	79 ± 6	0.421

Abbreviations: SBP, systolic blood pressure; DBP, diastolic blood pressure; TG, triglyceride; HDL-C, high-density lipoprotein cholesterol; FPG, fasting plasma glucose; LDL-C, low-density lipoprotein cholesterol; HbA1c, hemoglobin A1c; UA, uric acid; Ccr, creatinine clearance; VC, forced vital capacity; FEV1.0%, ratio of forced expiratory volume in 1 s

### Correlation between MetS factors and abnormal LVMR

We investigated the correlation between the abnormal values of the previously described MetS parameters, age, or BMI with abnormal LVMR. Univariate analysis showed that age and WC significantly correlated with abnormal LVMR in all healthy participants (odds ratio [OR] 1.099, 95% CI 1.079–1.119, *P* < 0.001; OR 1.04, CI 1.020–1.061, *P* < 0.001, respectively). In normal-weight participants, age, WC, and Ccr significantly correlated with abnormal LVMR (OR 1.086, 95% CI 1.057–1.115, *P* < 0.001; OR 2.735, CI 1.728–4.330, *P* < 0.001; OR 2.082, CI 1.211–3.578, *P* < 0.001, respectively).

### Multivariate analysis of MetS risk factors and LVMR

Next, we analyzed the risk factors for abnormal LVMR in relation to the metabolic risk factors. Because most cases of abnormal LVMR occurred in males, we analyzed the risk factors separately according to gender. In the multiple logistic analysis, only abnormal WC (≥85 cm) was an independent predictor of abnormal LVMR in men (OR 2.795, CI 1.649–4.737, *P* < 0.001). However, in women, both high DBP (≥85 mmHg) and abnormal WC (≥90 cm) were independent predictors of abnormal LVMR (OR 3.972, CI 1.557–10.136, *P* < 0.007; OR 2.086, CI 1.031–4.217, *P* = 0.041, respectively). In normal-weight subjects, aging, abnormal WC, and renal dysfunction (creatinine clearance < 60 mL/min) were independent predictors of abnormal LVMR (OR 1.080, CI 1.054–1.107, *P* < 0.001; OR 2.793, CI 1.793–4.352, *P* < 0.001; OR 2.142, CI 1.263–3.632, *P* = 0.005, respectively). In men, aging, abnormal WC (≥85 cm), and renal dysfunction were related to abnormal LVMR (OR 1.074, CI 1.041–1.109, *P* < 0.001; OR 3.698, CI 1.993–6.864, *P* < 0.001; OR 2.563, CI 1.218–5.391, *P* < 0.001, respectively). In contrast, in women, aging and high DBP (≥85 mmHg) were both significantly correlated with abnormal LVMR (OR 1.113, CI 1.074–1.115, *P* < 0.001; OR 3.976, CI 1.401–11.285, *P* = 0.010, respectively) (Table 4).

**Table 4:** Multivariate analysis of MetS risk factors and LVMR.

	Gender	MetS Factor	OR	95% C.I,	P value
All participants	Male	WC	2.795	1.649–4.737	< 0.001
	Female	DBP	3.972	1.557–10.136	0.007
		WC	2.086	1.031–4.217	0.004
Normal weight(BMI <25 kg/m^2^)	Male	Aging	1.074	1.041–1.109	< 0.001
		WC	3.698	1.993–6.864	< 0.001
		Renal dysfunction	2.563	1.218–5.391	< 0.001
	Female	Aging	1.113	1.074–1.115	< 0.001
		DBP	3.976	1.401–11.285	0.010

Abbreviations: OR, odds ratio; CI, confidence interval; WC, waist circumference; DBP, diastolic blood pressure

### Abnormal myocardial LVMR as a predictor of unplanned hospitalization

During a mean follow-up of 52 months, 1.5% (12/1055) of participants were hospitalized because of heart failure. All 12 had heart failure with preserved EF, and nine had an abnormal LVMR at the initial examination.

## Discussion

### Main findings

Our study revealed that in addition to aging, both abnormal WC and renal dysfunction were independent predictors of abnormal LVMR among normal-weight men, whereas only high DBP was an independent risk factor of abnormal LVMR in normal-weight women.

### Relationship between MetS and diastolic dysfunction

Although studies have been published on the relationship between MetS and diastolic dysfunction [11], none have identified potential predictors of LVMR by TDI. Recently, Russo et al. reported the correlation between BMI and E/e′ or e′ in adults [23]. In our study, although BMI showed a correlation with e′ by Spearman’s rank correlation coefficients, it was not correlated with abnormal LVMR in the univariate analysis and was not an independent predictor of LVMR when adjusted for age and gender in the multivariate analysis. We found that only abnormal WC was an independent predictor of abnormal LVMR in all healthy participants. Thus, we can assume that central adiposity is a significant factor for abnormal LVMR, particularly in healthy participants without obesity. It was recently reported that in individuals with metabolic disease, the presence of non-alcoholic fatty liver disease was independently associated with altered parameters of diastolic function [27]. These findings also support our results. Canepa et al. [17] have already shown the effect of central adiposity on LV diastolic function; however, in their study, the mean BMI was 27 ± 5 kg/m^2^ and the mean WC was 99 ± 10 cm in men and 85 ± 11 cm in women. This means that most study participants were overweight and had an abnormal waist circumference. Thus, it is assumed to be difficult to investigate the relationship between the MetS factors and LVMR. Hence, we investigated the relationship in healthy subjects without diabetes, and in those who were not overweight or obese.

### DBP was an independent risk factor of abnormal LVMR among women

In the present study, high DBP was an additional independent risk factor for abnormal LVMR among healthy women but not among healthy men. At least a partial explanation for this gender-specific result is the differences in vascular stiffness between women and men as a result of hormone release, such as estrogen [28, 29].

### Aging and central obesity in men

LVMR deteriorates with age and is a symptom of cardiac aging. Our results showed an increase in abnormal LVMR in an age-dependent manner (data not shown), and the average e′ also decreased with age, consistent with previous research [20]. We also found that WC and BMI correlated with LVMR. This indicates that the prevalence of obesity, a risk factor of diastolic LV dysfunction [23], is increased in elderly individuals. However, WC and not BMI was identified as an independent risk factor of abnormal LVMR in healthy men without obesity and overweight, suggesting that central adiposity is more important than obesity as a risk factor of abnormal LVMR.

### Extra-cardiac dysfunction and risk of abnormal LVMR

Previous studies have reported that higher serum Cr, lower FEV1 to FVC ratio, and lower Hb concentrations were associated with an increased risk of heart failure [30]. Our results indicated that only renal dysfunction was an independent risk factor of LVMR in healthy men. However, differences in study design preclude direct comparison and necessitate further investigation.

### Follow-up of abnormal LVMR

The Multi-ethnic Study of Atherosclerosis (MESA) reported that 0.3% of participants developed chronic heart failure during a 4-year follow-up. Those developing heart failure were more likely to be older, men, obese, current smokers, hypertensive, and diabetic [31]. Another cohort study reported that 2.6% of participants aged 45 years or older (n = 2042; mean age, 61.0 years; 49.4% men) had heart failure over a 6.3-year follow-up period. Additionally, this study reported that diastolic dysfunction was associated with incident heart failure [32]. Our results also suggest that abnormal LVMR could contribute to heart failure (with preserved LVEF), leading to unplanned hospitalization in participants without organic heart disease or diabetes mellitus.

### Clinical implication: the importance of WC in medical examinations

MetS is a cluster of atherogenic risk factors that includes abdominal obesity, insulin resistance, hypertension, dyslipidemia, disturbed pulmonary function, and a pro-inflammatory and pro-thrombotic state [33]. The results of our study showed that of the risk factors for MetS, only WC was an independent risk factor for abnormal LVMR in healthy participants. Doppler echocardiography is not included as a routine medical examination for LVMR screening; this study suggests that WC measurements can help select suitable patients for further LVMR assessment by Doppler echocardiography, without additional risk or cost.

### Study limitations

The strengths of our study included the use of detailed metabolic profiles, TDI assessment of diastolic dysfunction, and a high follow-up rate. However, this study also had several limitations. Participants were from urban areas in Japan, with a middle-class preponderance. Additionally, because our cohort was more than 99% Asian, the generalizability to other ethnic or racial populations may not be valid. In our study, we used the criteria for metabolic syndrome proposed by the Japanese Society of Hypertension and seven additional societies in 2005 [24]. Thus, additional research using the definitions of other local medical societies is necessary in order to determine if our findings apply to people of other ethnicities.

## Conclusion

Our retrospective cohort study demonstrated that the risk factors for LVMR varied according to gender. Among the MetS risk factors assessed in a thorough physical examination, only WC was found to be a useful predictor of diastolic dysfunction for healthy, normal-weight male participants, while DBP was found to a useful predictor of this condition in healthy, normal-weight female participants. Further prospective studies are necessary to verify our findings in different contexts.

## Supporting Information

S1 File Table A. The Total Health Care (THC) health check program.The table shows that the annual medical examinations of THC members. The left low shows the examination menu and the right low shows the detail of the examination. Fibrogastroscopy, Colonoscopy, and Head MRI and MRA are not included the annual medical examinations. These examination are optional menu with extra cost.Table B. Echocardiographic characteristics of the study cohort The table shows all echocardiographic data of the THC members. The left low shows the parameters of echocardiography. Data are presented as medians and interquartile range.
(DOCX)
