# Supplementary material for: Gender Differences in Predictors of Left Ventricular Myocardial Relaxation in Non-Obese, Healthy Individuals
Source: PLoS One. 2015 Apr 30;10(4):e0125107. doi: 10.1371/journal.pone.0125107 (PMC4416042; doi:10.1371/journal.pone.0125107)
Supplement: S1 File — Table A. The Total Health Care (THC) health check program.The table shows that the annual medical examinations of THC members. The left low shows the examination menu and the right low shows the detail of the examination. Fibrogastroscopy, Colonoscopy, and Head MRI and MRA are not included the annual medical examinations. These examination are optional menu with extra cost.Table B. Echocardiographic characteristics of the study cohort The table shows all echocardiographic data of the THC members. The left low shows the parameters of echocardiography. Data are presented as medians and interquartile range. (DOCX) [file pone.0125107.s001.docx]

**Table A. The Total Health Care (THC) health check program**

| Physical measurements | Height, weight, waist circumference, blood pressure, pulse rate |
| --- | --- |
| Laboratory measurements |  |
| Complete blood cell counts | WBC (Neut, Stab, Seg, Mono, Lymph, Eos), RBC (MCV, MCHC, MCH), Hb, Ht, Plt |
| Serum chemistry | TP, Alb, ZTT, TTT, AST, ALT, LDH, T-B, D-B, ALP, γ-GTP, ChE, Amy, LAP, BUN, Cr, Na, K, Cl, Ca, P, TC, TG, LDL-C, HDL-C, UA, FPG, HbA1c, insulin, hsCRP, Fe, |
|  | TSH, free T3, free T4, BNP |
| Urine analysis | pH, urine specific gravity, u-pro, u-glu, u-uro, u-keton, WBC, RBC |
| Tumor markers | CEA, CA19-9, CA-125, AFP, PSA |
| Chest, abdominal Xp |  |
| ECG |  |
| Head, chest, abdominal CT |  |
| Two-dimensional echocardiogram | AOD, LAD, IVST, PWT, LVDd, LVDs, FS, EF, E/A, DCT, e', E/e', RVSP |
| Carotid duplex |  |
| Abdominal ultrasound |  |
| ABI, PWV |  |
| Spirogram | VC, %VC, FEV1.0 |
| Gastrography |  |
| Eyesight test |  |
| Hearing test |  |
| Fibrogastroscopy | Optional |
| Colonoscopy | Optional |
| Head MRI and MRA | Optional |

Abbreviations: WBC, white blood cell; Neut, neutrophil; Seg, segment; Mono, mononucleosis; Lymph, lymphocyte; Eos, Eosinophil; RBC, red blood cell; MCV, Mean Corpuscular Volume; MCHC, mean corpuscular hemoglobin concentration; MCH, mean corpuscular hemoglobin; Hb, hemoglobin; Ht, hematocrit; Plt, platelet; TP, total protein; Alb, albumin; ZTT, zinc sulfate turbidity test; TTT, thymol turbidity test; AST, aspartate transaminase; ALT, aspartate aminotransferase; LDH, lactate dehydrogenase; T-B, total bilirubin; D-B, direct bilirubin; ALP, alkaline phosphatase; γ-GTP, γ-glutamyl transpeptidase; ChE, cholinesterase; Amy, amylase; LAP, leucine aminopeptidase; BUN, blood urea nitrogen; Cr, creatinine; Na, sodium; K, potassium; Cl, chloride; CA, calcium; P, phosphorus; TC, total cholesterol; TG, triglyceride; LDL-C, low-density lipoprotein cholesterol; HDL-C, high-density lipoprotein cholesterol; UA, uric acid; FPG, fasting plasma glucose; HbA1c, insulin, hemoglobin A1c; hsCRP, high sensitive C-reactive protein; Fe, iron; TSH, thyroid stimulating hormone; T3, Triiodothyronine; T4, thyroxin; BNP, brain natriuretic peptide; u-pro, urinary protein; u-glu, urinary glucose; u-uro, urinary urobilinogen; u-keton, ketone; CEA, carcinoembryonic antigen; AFP, alpha-fetoprotein; PSA, prostate specific antigen, AOD, aortic dimension; LAD, left atrial dimension; IVST, interventricular septum thickness; PWT, posterior LV wall thickness; LVDd, left ventricular diameter at end diastole; LVDs, left ventricular internal dimension in systole; FS, fractional shortening; EF, ejection fraction; E/A, peak early diastolic LV filling velocity/peak atrial filling velocity ratio; DCT, Deceleration time; E/e', the mitral early diastolic peak flow velocity (E) to tissue Doppler early mitral annular diastolic velocity (e´) ratio; RVSP, right ventricular systolic pressure; ABI, ankle brachial index; PWV, pulse wave velocity; VC, forced vital capacity; FEV1.0%, ratio of forced expiratory volume in 1 s

**Table B. Echocardiographic characteristics of the study cohort**

|  | All participants | | | Normal weight  (BMI <25 kg/m^2^) | | |
| --- | --- | --- | --- | --- | --- | --- |
|  | All  (n = 1055) | Men  (n = 620) | Women  (n = 435) | All  (n = 806) | Men  (n = 421) | Women  (n = 385) |
| AOD (mm) | 32 (28-35) | 34 (31-36) | 29 (27-31) | 31 (28-34) | 33 (31-36) | 28 (26-31) |
| LAD (mm) | 34 (30-37) | 35 (32-38) | 31 (28-34) | 32 (29-36) | 34 (31-37) | 31 (28-34) |
| IVST (mm) | 9 (8-10) | 10 (9-11) | 9 (8-9) | 9 (8-10) | 10 (9-11) | 8 (8-9) |
| PWT (mm) | 9 (8-10) | 10 (9-10) | 8 (7-9) | 9 (8-10) | 9 (9-10) | 8 (7-9) |
| LVDd (mm) | 45 (42-49) | 47 (44-50) | 43 (41-45) | 48 (45-52) | 47 (44-50) | 43 (40-45) |
| FS | 37 (35-40) | 37 (34-39) | 38 (35-41) | 37 (35-40) | 37 (34-39) | 38 (35-41) |
| EF (%) | 68 (64-71) | 67 (63-70) | 69 (65-72) | 68 (64-71) | 67 (63-70) | 69 (65-72) |
| E/A ratio | 0.9 (0.8-1.1) | 0.9 (0.7-1.1) | 1.0 (0.8-1.2) | 0.9 (0.8-1.2) | 0.9 (0.7-1.1) | 1 (0.8-1.3) |
| DCT(s) | 229 (196-262) | 236 (203-272) | 214 (186-249) | 225 (193-257) | 234 (201-269) | 214 (186-247) |
| eʹ | 6.8 (5.4-8.2) | 6.6 (5.3-7.7) | 7.1 (5.6-9.0) | 7.0 (5.6-8.5) | 6.8 (5.4-7.9) | 7.3 (5.8-9.3) |
| E/eʹ | 9.1 (7.4-11.2) | 9.0 (7.3-11.1) | 9.4 (7.5-11.4) | 9.0 (7.3-11.0) | 8.8 (7.1-10.7) | 9.2 (7.5-11.3) |
| TR (cm/s) | 2.2 (2.0-2.4) | 2.2 (2.0-2.4) | 2.2 (2.0-2.4) | 2.2 (2.0-2.4) | 2.2 (2.0-2.4) | 2.2 (2.0-2.4) |
| RVSP (mmHg) | 29.0 (26.4-32.3) | 28.8 (26.3-32.3) | 29.3 (26.5-32.0) | 29.3 (26.5-32.3) | 29.1 (26.4-32.5) | 29.4 (26.5-31.7) |
| LVMI (51 g/m^2.7^) | 41.4 (34.1-49.8) | 44.5 (36.4-51.9) | 38.1 (31.4-45.4) | 39.8 (32.3-47.0) | 42.4 (35.3-49.8) | 37.0 (30.8-42.2) |
| RWT | 0.40 (0.36-0.44) | 0.41 (0.37-0.45) | 0.39 (0.35-0.43) | 0.40 (0.36-0.43) | 0.40 (0.37-0.44) | 0.38 (0.35-0.42) |

Abbreviations: AOD, aortic dimension; LAD, left atrial dimension; IVST, interventricular septum thickness; PWT, posterior LV wall thickness; LVDd, left ventricular diameter at end diastole; LVDs, left ventricular internal dimension in systole; FS, fractional shortening; EF, ejection fraction; E/A, peak early diastolic LV filling velocity/peak atrial filling velocity ratio; DCT, Deceleration time; E/e', the mitral early diastolic peak flow velocity (E) to tissue Doppler early mitral annular diastolic velocity (e´) ratio; TR, Tricuspid Regurgitation; RVSP, right ventricular systolic pressure, LVMI, left ventricular mass index; RWT, relative wall thickness
